# Multi-drug-resistant enterococcal infections: duration of combination therapy and OPAT challenges

**DOI:** 10.1128/aac.01753-25

**Published:** 2026-03-16

**Authors:** Bryan P. White, Emily A. Siegrist

**Affiliations:** 1 Department of Pharmacy, University of Oklahoma Medical Center 6186, Oklahoma City, Oklahoma, USA; University of California San Francisco, San Francisco, California, USA

**Keywords:** daptomycin, ampicillin, OPAT, VRE

## LETTER

We read with interest the paper by Turner et al. reviewing the treatment of difficult-to-treat multi-drug-resistant enterococcal infections (1). We applaud them for this comprehensive review of a common, challenging clinical scenario. We have some comments on the duration of combination therapy and difficulties of combination therapy with outpatient parenteral therapy (OPAT).

For high-risk *Enterococcus faecium* infections, Turner et al. recommend combination therapy with daptomycin plus ampicillin as first-line therapy. This is based on retrospective clinical data and *in vitro* studies which support possibly improved patient outcomes and synergy when using beta-lactam and daptomycin combination therapy. The optimal duration of this combination therapy and when to step down to monotherapy is not clear, but combination therapy may not be needed for the whole course. The largest clinical paper that they cite for this recommendation only used beta-lactams (median duration of 9 days, IQR 4–13) for part of the course of daptomycin (median duration of 13 days, IQR 8–15) (Y. Chuang, personal communication, September 2025) (2). The *in vitro* data reviewed consisted of modeled infections for 4 (3) and 14 days (4), which would not support dual therapy duration for longer than these time periods. Clinical data from MRSA bacteremia patients using ceftaroline in combination with daptomycin showed no difference in outcomes when patients were deescalated to monotherapy before 10 days or completed therapy with combination therapy (5). In contrast, combination therapy with ampicillin and ceftriaxone is recommended for the whole course of *Enterococcus faecalis* endocarditis, but there are no standard recommendations for complicated bacteremia in the absence of endocarditis (6).

Given the prolonged course necessary when treating enterococcal infections like endocarditis or complicated bacteremia, long-term dual therapy with daptomycin and a beta-lactam may result in significant logistical issues, particularly patients discharging on OPAT (6). Ampicillin has a short stability once reconstituted and is stable for only 72 h when stored at 4°C (7). This necessitates multiple times weekly drug delivery, which is not possible in many areas. Mini-bag administration with ampicillin is possible, but requires patients to mix and self-administer ampicillin as frequently as every 4 h. With limited clinical data and logistical challenges, combination therapy for *E. faecium* infections may not be necessary for the whole treatment course.
